# Current News

**Published:** 1895-01

**Authors:** 


### Current News.

### CONNECTICUT VALLEY DENTAL SOCIETY.

At the Thirty-first Annual Meeting of the Connecticut Valley Dental Society, held at Springfield, Mass., October 25 and 26, the following officers were elected for the ensuing year:

President, Dr. C. C. Barker, Meriden, Conn.; First Vice-President, Dr. W. O. Barrett, Ware, Mass.; Second Vice-President, Dr. D. A. Jones, New Haven, Conn.; Secretary, Dr. George A. Maxfield, Holyoke, Mass.; Assistant Secretary, Dr. A. J. Cutting, Southington, Conn.; Treasurer, Dr. F. R. Rice, North Adams, Mass.

Geo. A. Maxfield, D.D.S.,
*Secretary.*

### WOMAN'S DENTAL ASSOCIATION.

The Woman's Dental Association met at Dr. Maria Lasser's office, 1602 Arch Street, Philadelphia, Pa., November 3, 1894.

Vice-President Dr. Elizabeth A. Davis in the chair.

Dr. J. Foster Flagg discussed Dr. Mascort's article, published in the *Dental Cosmos* for May, on "Salol in Filling Roots."

The next meeting will be held at Dr. Yerkes's office, 4004 Chestnut Street, December 1, 1894.

Emily W. Wyeth,
*Recording Secretary.*

### RECENT PATENTS.

A LIST of recent patents reported for the INTERNATIONAL DENTAL JOURNAL:

523,049.—Adjustable Chair. Joseph R. Miller, Erie, Pa., assignor of one-half to J. R. Phillips, same place. Filed Feb. 24, 1893.

523,192.—Tooth-Regulator. Edward H. Angle, Minneapolis, Minn. Filed May 7, 1894.

523,472.—Method of Forming Tooth-Crowns. Jephtha G. Hollingsworth, Kansas City, Mo. Filed May 3, 1894.

524,419.—Dental Mouth-Mirror. Alfred E. Gray, Uxbridge, Mass. Filed June 1, 1894.

524,798.—Dental Vulcanizer. James H. Beebee, Rochester, assignor to the Buffalo Dental Manufacturing Company, Buffalo, N. Y. Filed May 4, 1894.

525,051.—Adjustable Chair. Charles H. Knight, Rondout, and Lemuel A. Chichester and David S. Relyea, Chichester, assignors to the National Chair Manufacturing Company, Chichester, N. Y. Filed June 14, 1893.

525,166.—Dental or Surgical Chair. Edmund P. Stiles, Austin, Tex. Filed June 3, 1891.

525,278.—Dental Tool-Guard. Arthur E. Peck, Minneapolis, Minn. Filed July 29, 1893.

Trade-Marks.—25,053.—Toothache Remedy. Harle, Haas & Co., Council Bluffs, Iowa. Filed February 25, 1893. Essential feature, the word "Magic."

